# Enhancing predictive modeling for respiratory support with LLM-driven guideline adherence

**DOI:** 10.1186/s13054-025-05739-3

**Published:** 2025-11-14

**Authors:** Xiaolei Lu, Michael Miller, Alex K. Pearce, Preeti Gupta, Thaidan T. Pham, James Ford, Atul Malhotra, Shamim Nemati

**Affiliations:** 1 Department of Biomedical Informatics, University of California, San Diego, La Jolla, CA USA; 2 Division of Pulmonary, Critical Care, and Sleep Medicine, University of California, San Diego, La Jolla, CA USA; 3 9500 Gilman Drive MC 0881, La Jolla, CA 92093 USA

**Keywords:** Causal inference, Large language models, Individualized treatment effect, High-flow nasal cannula, Noninvasive ventilation, Guideline adherence

## Abstract

**Background:**

Optimal respiratory support selection between high-flow nasal cannula (HFNC) and noninvasive ventilation (NIV) for intensive care units (ICU) patients at risk of invasive mechanical ventilation (IMV) remains unclear, particularly in cases not represented in prior clinical trials. We previously developed RepFlow-CFR, a deep counterfactual model estimating individualized treatment effects (ITE) of HFNC versus NIV. However, interpretability and guideline alignment remain challenges for clinical adoption. This study describes the development and integration of a clinical guideline-driven LLM to enhance deep counterfactual model recommendations for NIV versus HFNC in patients at high-risk for invasive mechanical ventilation.

**Methods:**

We enhanced RepFlow-CFR by incorporating a large language model (LLM, Claude 3.5 Sonnet) to enforce clinical guideline adherence and generate explainable treatment recommendations. The LLM was configured in a HIPAA-compliant AWS environment and prompted using structured patient data, clinical notes, and formal guideline criteria. Recommendations from RepFlow-CFR and LLM were compared to actual treatment decisions to assess concordance. We evaluated IMV and mortality/hospice rates across concordant and discordant groups. Additionally, we conducted a structured chart review of 20 cases to assess the clinical validity and safety of LLM-driven recommendations.

**Results:**

Among 1,261 ICU encounters, treatments concordant with LLM-enhanced recommendations were associated with lower IMV rates. For the HFNC recommendation, IMV occurred in 46/188 (24.47%) when care was concordant versus 9/17(52.94%) when discordant, corresponding to a 97.33% relative risk increase when discordant. Concordance was also associated with reduced mortality or hospice discharge (odds ratio 0.670, *p* = 0.046). In a 20-case chart review, 19/20 (95%) LLM recommendations aligned with clinical guidelines and physicians agreed with 13/20 (65%) final recommendations. Errors were noted in 11/20 cases, most rated low or moderate risk; 2/20 were judged as potentially causing severe harm.

**Conclusions:**

Integrating LLMs for guideline enforcement improves the interpretability and clinical alignment of counterfactual models in respiratory support decision-making. This hybrid framework not only enhances concordance with real-world practice but may also improve patient outcomes. Future work will refine contraindication detection and expand validation to prospective clinical trials.

**Supplementary Information:**

The online version contains supplementary material available at 10.1186/s13054-025-05739-3.

## Background

Acute respiratory failure (ARF) is common in critically ill patients, affecting about half of all intensive care units (ICU) admissions. Among patients not requiring urgent intubation and invasive mechanical ventilation (IMV), high-flow nasal cannula (HFNC) and noninvasive ventilation (NIV) are commonly used respiratory support therapies [1–6]. The choice of initial respiratory support therapy can impact important patient outcomes like need for IMV or mortality, however, the choice based on prior randomized controlled trials (RCTs). Patients can have overlapping clinical indications for both HFNC or NIV, thus, there is considerable clinical equipoise when choosing between these treatment modalities in real-time. Furthermore, many patients with ARF in the real-world setting who receive HFNC and NIV would not fit the inclusion and exclusion criteria of previously conducted comparing the two modalities. There is also the possibility of heterogeneity of treatment effect, in which baseline patient characteristics influence response to therapy. The uncertainty introduced by these features highlights the need for data-driven precision medicine techniques to assist with the choice of NIV or HFNC [8, 9].

Machine learning techniques can be employed to estimate the individualized treatment effect (ITE) of high-flow nasal cannula (HFNC) versus noninvasive ventilation (NIV) for each patient [7]. Recent advancements in causal inference and counterfactual modeling have produced a diverse toolkit for ITE estimation in observational data. These tools include tree-based methods (e.g., Causal Forests [12]), representation learning approaches (e.g., TARNet [13]), meta-learners (e.g., X-learner [14]), and balancing techniques such as Bayesian Additive Regression Trees [15]. Building upon this foundation, we previously developed and validated RepFlow-CFR, a deep counterfactual representation model tailored to the critical care setting. RepFlow-CFR is designed to estimate patient-specific outcomes under both HFNC and NIV by learning balanced representations that adjust for confounding and capture latent patient heterogeneity. The model combines shared representation learning, normalizing flows for outcome modeling, and a second-stage adjustment for unmeasured confounding, enabling it to generate robust, individualized predictions for response to NIV versus HFNC, thereby helping inform treatment decisions that decrease the risk of IMV. When applied to ICU patients identified as high-risk for respiratory failure, patients receiving treatments concordant with RepFlow-CFR recommendations showed lower rates of IMV and mortality or hospice discharge, highlighting the model’s ability to identify beneficial therapy pathways in complex ICU populations.

While RepFlow-CFR demonstrated strong performance in generating individualized treatment recommendations and identifying beneficial therapy pathways, it shares a common limitation with many deep learning models, namely, a lack of transparency in its decision-making process. This “black box” nature makes it difficult for clinicians to interpret or trust the model’s output, especially when recommendations diverge from established clinical guidelines. Moreover, RepFlow-CFR is not inherently constrained to follow these guidelines, which may lead to discordance between data-driven recommendations and evidence-based best practices. To address these challenges, we introduce a large language model (LLM) into the decision pipeline to serve as a guideline-aware, explainable reasoning layer. By leveraging structured patient data, clinical notes, and formal guideline criteria, the LLM can validate or refine the model’s recommendations, ensuring that treatment suggestions align with current standards of care while also providing interpretable justifications. In this study, we aim to refine treatment recommendations from a deep counterfactual model by integrating LLM to enforce adherence to clinical guidelines and enhance interpretability. We further evaluate the impact of this LLM-augmented framework on concordance with real-world treatment decisions and patient outcomes, with additional validation through structured expert chart review. We hypothesized that integrating a LLM with a deep counterfactual model to refine treatment recommendations would enhance interpretability, result in treatment suggestions that better align with established guideline driven standard of care.

## Methods

### RepFlow-CFR prediction

**Plain-language summary of the model.** Our aim is to estimate, for each patient, whether NIV or HFNC is more likely to avoid IMV. We first learn a patient summary that balances measured factors between treatment groups (analogous to propensity-score balancing). We then use a reversible (“invertible”) neural network—formally, a conditional normalizing flow (CNF)—to learn how outcomes are distributed given that summary and the treatment. Finally, we include an adjustment step for residual (unmeasured) bias so the resulting patient-specific risk estimates and recommendations are more clinically reliable.

**Fig. 1:**
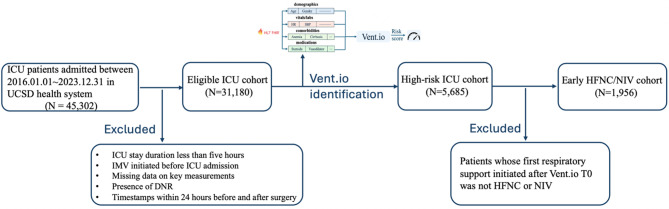
Cohort selection flowchart for early HFNC/NIV analysis

**Data sources.** We used de-identified structured Electronic Health Record (EHR) data from ICU patients at UC San Diego Health (UCSD) between January 1, 2016, and December 31, 2023, focusing on encounters where either HFNC or NIV was administered as the first respiratory support following Vent.io-predicted [16] high-risk timepoint (Vent.io T0). Figure 1 illustrates the cohort derivation process. Extracted variables included 50 vital signs and laboratory measurements (resampled into hourly bins), 6 demographic features, 12 SIRS/SOFA criteria, 12 medication categories, and 62 comorbidities. Additional derived features included baseline values, local trends, and time since last measurement (TSLM). Missing values were forward-filled up to 24 h or mean-imputed (Supplementary Sect. 1).

**RepFlow-CFR model.** The RepFlow-CFR model is a deep counterfactual inference framework that adjusts for confounding using both observed and inferred latent variables. It consists of three stages (Supplementary Sect. 2 for the model architecture and mathematical formulation): **Stage 0** applies counterfactual regression (CFR) [13] to learn shared representations balancing measured confounders across treatment groups using an integral probability metric (e.g., Wasserstein distance); **Stage 1** uses a conditional normalizing flow (CNF) [17] to model the outcome distribution given the representation and treatments; **Stage 2** introduces a second CNF to adjust for unmeasured confounding by transforming the treatment-dependent latent variable into an interventional distribution.

**Model training and evaluation.** The UCSD HFNC/NIV cohort was randomly divided into 80% training and 20% validation. The validation set was used exclusively for hyperparameter tuning and early stopping. Hyperparameters were tuned via Bayesian optimization across learning rate, weight regularization, number of hidden layers, flow depth, dropout rate, and batch size. Each module (CFR backbone and both CNFs) was trained separately with the Adam optimizer, with early stopping applied to mitigate overfitting.

During inference, RepFlow-CFR generated potential outcomes under both HFNC and NIV by sampling 100 latent draws per encounter from the Stage 2 CNF and mapping them through Stage 1. Predicted outcomes were then averaged to produce stable estimates for each treatment condition. The individual treatment effect (ITE) was defined as the difference in predicted probabilities of IMV under NIV versus HFNC. We adopt a small indifference band for ITE: NIV preferred if ITE < − 0.001, HFNC preferred if ITE > 0.001, otherwise *Indifferent*. This band buffers near-zero estimation noise and discourages acting on ≤ 0.1-percentage-point risk differences.

After hyperparameter tuning, the optimized model (trained on the 80% subset) was applied to the entire UCSD cohort to generate ITEs for descriptive cohort-level analyses. Performance was assessed using predictive discrimination (AUC, PR-AUC). On the UCSD cohort, RepFlow-CFR achieved an AUC of 0.820 and PR-AUC of 0.566. The complete hyperparameter grid is provided in Supplementary Sect. 2.

### LLM-driven guideline enforcement

#### Claude sonnet configuration and deployment

We utilized Claude 3.5 Sonnet [19], a large language model with a 200,000-token context window, optimized for high-accuracy reasoning and structured clinical analysis. The model was deployed within a HIPAA-compliant Amazon Web Services (AWS) environment using an EC2 instance configured with authenticated access to Amazon Bedrock. Inference was performed via the Bedrock Converse API, which supports structured JSON output for reliable parsing. To minimize variability and ensure consistent, deterministic responses, the model operated with a temperature setting of 0.1. Claude Sonnet was used to evaluate whether RepFlow-CFR recommendations were aligned with clinical guidelines and to independently generate treatment recommendations based on structured patient data, clinical notes and formal guideline criteria.

#### Clinical guidelines criteria

We summarize the recommended indications for NIV and HFNC based on established clinical guidelines. Specifically, we refer to the ERS/ATS 2017 Guidelines for NIV [11] and the ERS 2022 Guidelines for HFNC [10]. The guideline criteria are outlined in Table 1 below, highlighting when each therapy is recommended, conditionally recommended, or not advised.

**Table 1:** Clinical indications for NIV and HFNC according to ERS/ATS guidelines

NIV – ERS/ATS 2017 guidelines Recommend NIV if any of the following are true:	HFNC – ERS 2022 guidelines Recommend HFNC if any of the following are true:
– COPD exacerbation with acute or acute-on-chronic respiratory acidosis (pH ≤ 7.35, PaCO₂ >45 mmHg)	– De novo (or acute) hypoxemic respiratory failure (e.g., AHRF, ARDS, pneumonia, COVID-19)
– Acute respiratory failure due to cardiogenic pulmonary edema, including acute (or “flash”) pulmonary edema	– Post-operative post-extubation in high-risk patients: HFNC is preferred over conventional oxygen but NIV is also an acceptable prophylactic treatment for high-risk patients
– Neuromuscular disease or obesity hypoventilation syndrome (OHS) with acute or acute-on-chronic respiratory acidosis (pH ≤ 7.35, PaCO₂ >45 mmHg)	– The patient is intolerant to NIV and moderate to severe acute respiratory failure is present
– Use NIV prophylactically post-extubation in high-risk patients (e.g., those with COPD or CHF) but not in low-risk patients or in patients with established post-extubation respiratory failure	– Moderate to severe acute respiratory failure is present and there is not an indication for treatment with NIV
– Immunocompromised patients with mild-to-moderate acute respiratory failure (conditional recommendation)	Do not recommend HFNC as first-line if: – The patient has acute hypercapnic respiratory failure (e.g., COPD with acidosis) unless NIV is contraindicated or not tolerated – There is clear guideline indication for NIV
– Post-operative ARF (Conditional recommendation, moderate certainty of evidence)	
– Chest trauma patients with ARF (Conditional recommendation, moderate certainty of evidence)	

#### LLM-Assisted guideline concordance assessment

**Inputs to LLM**: To generate explainable, guideline-aligned recommendations, the LLM consumed a structured bundle comprising: (i) summarized indications/contraindications from ERS/ATS 2017 and ERS 2022 guidelines; (ii) the Vent.io T0 timestamp (first high-risk flag); and (iii) RepFlow-CFR outputs at T0: the provisional modality (NIV/HFNC/Indifferent) plus a rationale represented by the top-50 SHAP-ranked features (chosen a priori for interpretability). These inputs allow the LLM to check eligibility/contraindications, audit or correct the provisional choice, and produce a concise patient-specific rationale. Table 2 details the input schema.

**Table 2:** Inputs provided to the LLM

Input type	Description
**Summarized clinical guidelines**	Guideline-based indications and contraindications for NIV and HFNC (from Sect. Clinical guidelines criteria), enabling the LLM to reason using ERS/ATS 2017 and ERS 2022 recommendations.
**Vent.io T0**	Timestamp indicating when the patient was first identified as high-risk for respiratory failure by the Vent.io early warning model.
**RepFlow-CFR model output at T0**	Includes (1) the recommended treatment modality (NIV, HFNC, or Indifferent), and (2) the model rationale based on EHR-derived features. The rationale includes the top 50 most influential SHAP-ranked features impacting the decision.
**Recent clinical parameters (pre-T0)**	Most recent values prior to T0 for: pH, PaCO₂, respiratory rate, SpO₂, and FiO₂.
**Relevant clinical notes (≤ 72 h pre-T0)**	Free-text notes extracted from the EHR within 72 h before T0, including: ED provider notes, ED MD progress notes, consults, ED OBS HPI, progress notes, H&P, Event/Update entries, ED OBS progress notes, and radiology reports.

**Prompting strategy**: We instructed the LLM to (i) assess concordance between the RepFlow-CFR recommendation and guideline-based criteria derived from the patient record, (ii) issue an independent recommendation: NIV, HFNC, or Indifferent and (iii) justify the decision by explicitly citing the relevant guideline statements. Full details, including context ordering, JSON schema, and safeguards to reduce unsupported outputs, are provided in Supplementary Section S3.

##### Output of the LLM

The LLM was prompted to return a structured JSON object as listed in Table 3.

**Table 3:** Structured output returned by the LLM

Key	Value/Format
**“NIV_recommendation”**	{ “recommendation”: “Yes” or “No” or “Either”, “confidence”: “high”/“medium”/“low”, “explanation”: “Brief rationale including guideline Reference” }
**“HFNC_recommendation”**	{ “recommendation”: “Yes” or “No” or “Either”, “confidence”: “high”/“medium”/“low”, “explanation”: “Brief rationale including guideline Reference” }
**“Model_alignment”**	{ “alignment”: true/false, “explanation”: “Was the RepFlow-CFR model aligned with guideline-based decision?” }

### Concordance analysis

We assessed the concordance between model-generated treatment recommendations and actual clinical decisions at two levels. First, RepFlow-CFR Concordance was defined as agreement between the treatment modality recommended by the RepFlow-CFR model: NIV, HFNC, or Indifferent, and the treatment the patient actually received immediately following the Vent.io T0 timepoint. Second, LLM-Enhanced Concordance was defined as agreement between the final treatment recommendation generated by the LLM, which integrated clinical guidelines and patient-specific data, and the actual treatment administered. For both types of recommendations, we compared key clinical outcomes between concordant and discordant groups, specifically focusing on the incidence of IMV and a composite endpoint of in-hospital mortality or discharge to hospice (Mortality/Hospice).

### Chart review validation

To assess the clinical validity and safety of the LLM-enhanced recommendation framework, we conducted a retrospective chart review of 20 patient cases. These cases were selected to represent a variety of clinical scenarios, including variations in disease severity, instances where the LLM and the RepFlow-CFR model generated discordant treatment recommendations. This purposive sampling strategy was designed to challenge the LLM’s interpretive and decision-making capacity across a spectrum of relevant clinical contexts.

Each case was independently reviewed by three board-certified critical care physicians. Reviewers evaluated the LLM’s treatment recommendation for congruence with established clinical guidelines, specifically the ERS/ATS 2017 guideline for NIV and the ERS 2022 guideline for HFNC. In addition to assessing whether the LLM’s final recommendation aligned with guideline-based care, the reviewers also examined the explanation provided by the LLM, with particular attention to the presence of factual errors, clinically significant omissions, or hallucinated statements.

To evaluate the LLM’s comprehension, knowledge retrieval, and reasoning ability, a structured evaluation framework was used, adapted from prior work in validating medical LLM outputs [18]. Given the known tendency of LLMs to mix correct and incorrect content, the review framework was designed to explicitly capture both accurate and inaccurate components of the model’s response. To assess the potential for harm, the reviewers used the harm classification framework from the Agency for Healthcare Research and Quality (AHRQ) Common Formats.

The structured evaluation criteria used by physician reviewers are summarized in Table 4. These include assessments of recommendation congruence, explanation accuracy, potential for harm, clinical agreement, and LLM-specific reasoning and retrieval performance. Each criterion was accompanied by categorical evaluation options and a space for free-text reviewer comments.

**Table 4:** Structured evaluation criteria for LLM chart review

Domain	Evaluation criteria	Options/Scale	Reviewer comments
**Recommendation congruence**	Is recommendation congruent with guideline?	Yes/No	
**Explanation accuracy**	Is there incorrect content in the explanation?	Yes/No	
	Is the incorrect explanation clinically significant?	Yes/No	
	Is the explanation missing important clinical content?	Yes/No	
**Error harm assessment**	Harm Likelihood for LLM error	Low/Medium/High/N.A.	N.A. was put in for the cases where there was no LLM error
	Harm Extent from LLM error	None/no harm, Mild/Moderate, Severe/death, NA	N.A. was put in for the cases where there was no LLM error
**Clinical judgment**	Overall MD agreement with LLM recommendation	Agree/Disagree/Partially Agree	
	Second MD agreement (if any)	Agree/Disagree/Partially Agree	
**LLM comprehension & reasoning**	Is comprehension of the question correct?	Yes/No	
	Is evidence retrieval correct?	Yes/No	
	Is reasoning for LLM recommendation correct?	Yes/No/Partially	
	Any incorrect comprehension from LLM?	Yes/No	
	Any incorrect or missing clinical info (retrieval error or hallucination)?	Yes/No	
	Did the LLM recommendation contain incorrect rationale?	Yes/No	

## Results

### Patient characteristics, interventions, and outcomes by RepFlow-CFR and LLM-enhanced recommendation

Our study cohort included 1,261 ICU encounters, stratified by treatment recommendations from the RepFlow-CFR and LLM-enhanced models, as shown in Table 5. RepFlow-CFR classified 891 patients as NIV-preferred, 252 as HFNC-preferred, and 118 as indifferent, while the LLM-enhanced model reassigned 759, 205, and 297 patients, respectively. Baseline characteristics were generally balanced: mean age ranged from 60 to 63 years, the proportion of male patients from 57.6% to 59.9%, and SOFA scores at Vent.io T0 were similar across groups.

Post-T0 interventions including steroids, antibiotics, vasopressors, and diuretics did not differ significantly. Concordance with recommendations was higher for the LLM model, with 91.7% of HFNC-preferred patients receiving HFNC compared to 68.3% under RepFlow-CFR. Clinical outcomes were also similar across groups: IMV occurred in 26–29% of patients, mortality ranged from 24.4% to 41.8%, and hospice enrollment was < 1.2%, with no statistically significant differences observed. These findings suggest that measured confounding was well balanced, while the LLM-enhanced model improved alignment between recommendations and observed clinical practice.

**Table 5:** Baseline characteristics of patients by RepFlow-CFR and LLM-enhanced recommendations

Variable	RepFlow-CFR recommendation				LLM-enhanced recommendation			
	NIV preferred	HFNC preferred	Indifferent	*P*^b^ value	NIV preferred	HFNC preferred	Indifferent	*P* value
**Characteristic**								
Encounters, N	891	252	118	-	759	205	297	-
Age(years), mean (SD)	62(16.7)	61(16.4)	63(15.7)	0.580	63(16.4)	60(18.0)	60(15.5)	0.580
**Gender**,** N (%)**								
Male	516(57.9)	151(59.9)	70(59.3)	-	442(58.2)	118(57.6)	177(59.6)	-
**Organ dysfunction**								
**Charlson comorbidity index**,** median (IQR)**	2.0 (1.0–4.0)	2.0 (1.0–5.0)	3.0 (1.0–6.0)	0.361	3.0(1.0–5.0)	1.0(1.0–3.0)	2.0(2.0–6.0)	0.361
Congestive heart failure component, N (%)	208(23.3)	63(25.0)	41(34.7)	0.800	261(34.4)	18(8.8)	33(11.1)	0.800
Chronic obstructive pulmonary disease, N (%)	166(18.6)	47(18.7)	24(20.3)	0.996	176(23.2)	28(13.7)	33(11.1)	0.996
SOFA score (at Vent.io T0), median (IQR)	1.0(0.0–3.0)	1.0(0.0–3.0)	1.0(0.0–3.0)	0.512	2.0(0.0–3.5.0.5)	0.0(0.0–2.0)	1.0(0.0–3.0)	0.512
**Interventions after Vent.io T0**^**a**^ **N (%)**								
Steroids administration	301(33.8)	71(28.2)	39(33.1)	0.129	225(29.6)	65(31.7)	121(40.7)	0.129
Antibiotics administration	744(83.5)	211(83.7)	93(78.8)	0.154	627(82.6)	154(75.1)	267(89.9)	0.154
Vasopressors administration	273(30.6)	78(31.0)	33(28.0)	0.306	276(36.4)	39(19.0)	69(23.2)	0.306
Diuretics administration	526(59.0)	144(57.1)	79(66.9)	0.424	462(60.9)	118(57.6)	169(56.9)	0.424
**Initial respiratory support after Vent.io T0 (N %)**								
HFNC	671(75.3)	172(68.3)	80(67.8)	-	485(63.9)	188(91.7)	250(84.2)	-
NIV	220(24.7)	80(31.7)	38(32.2)	-	274(36.1)	17(8.3)	47(15.8)	-
**Outcomes (N %)**								
IMV	252(28.3)	70(27.8)	17(14.4)	0.159	197(26.0)	55(26.8)	87(29.3)	0.159
Mortality	318(35.7)	72(28.6)	37(31.4)	0.977	253(33.3)	50(24.4)	122(41.8)	0.977
Hospice	8(0.9)	3(1.2)	0(0.0)	0.480	8(1.1)	2(1.0)	1(0.3)	0.480

^a^We focused on interventions administered from Vent.io T0 to one hour before ICU discharge for the control group (those not intubated), and from Vent.io T0 to one hour before the time of intubation for the positive group (those who require intubation)

^b^Testing for the difference *P *value were χ2 for categorical variables and Kruskal-Wallis rank-sum test for continuous variables

### Concordance analysis

To evaluate the clinical utility of model-guided recommendations, we analyzed the concordance between predicted treatment recommendations and the actual treatments received by patients, stratified by outcome. The primary outcome was the need for invasive mechanical ventilation (IMV); mortality or hospice discharge was assessed as a secondary outcome.

Tables 6 and 7 report IMV and mortality/hospice rates among patients whose received treatment was concordant versus discordant with the model recommendation. To quantify effect direction and magnitude, we compute relative change against the recommendation-specific total outcome rate: relative reduction if concordant = ((Total IMV − Concordant IMV)/Total IMV) × 100%; relative increase if discordant = ((Discordant IMV − Total IMV)/Total IMV) × 100%. Across both models, patients who received treatments concordant with model recommendations had lower IMV rates. The LLM-enhanced model demonstrated greater relative reductions in IMV rates in the concordant groups, particularly under HFNC recommendations, where discordant treatment was associated with a 97% relative increase in IMV risk. For the mortality and hospice outcome, the LLM-enhanced model again showed larger differences between concordant and discordant groups. Under the LLM framework, NIV-concordant cases showed a 14.03% relative reduction in mortality/hospice, compared to only 6.83% under the baseline model.

We further assessed the association between concordant treatment and outcomes using multivariable logistic regression, controlling for potential confounders including age, gender, CCI, SOFA score, and Vent.io risk score. The results are summarized in Table 8. Within the LLM-enhanced framework, concordance was associated with lower odds of both IMV and Mortality/Hospice, with heterogeneity by recommended-modality stratum. For HFNC-recommended patients, concordant care was linked to lower odds of Mortality/Hospice (OR = 0.67, 95% CI 0.45–0.99; *p* = 0.046). Given multiple subgroup contrasts and some borderline p-values, we emphasize effect estimates with 95% CIs and treat these analyses as exploratory.

**Table 6:** IMV rates stratified by model recommendation and concordance (%)

Model	Recommendation	Total IMV	Concordant IMV	Discordant IMV	Relative reduction if concordant	Relative increase if discordant
**RepFlow-CFR**	**NIV**	28.28 (172/891)	22.73 (50/220)	30.10 (202/671)	19.64	6.44
	**HFNC**	27.78 (70/252)	25.00 (43/172)	33.75 (27/80)	10.00	21.50
**LLM-enhanced**	**NIV**	25.96 (197/759)	21.17 (58/274)	28.65 (139/485)	18.44	10.42
	**HFNC**	26.83 (55/205)	24.47 (46/188)	52.94 (9/17)	8.80	97.33

**Table 7:** Mortality & hospice rates stratified by recommendation and concordance (%)

Model	Recommendation	Total mortality & hospice	Concordant mortality & hospice	Discordant mortality & hospice	Relative reduction if concordant	Relative increase if discordant
**RepFlow-CFR**	**NIV**	36.59 (326/891)	34.09 (75/220)	37.41 (251/671)	6.83	2.24
	**HFNC**	29.76 (75/252)	30.23 (52/172)	28.75 (23/80)	−1.58	−3.40
**LLM-enhanced**	**NIV**	34.39 (261/759)	29.56 (81/274)	37.11 (180/485)	14.03	7.93
	**HFNC**	25.37 (52/205)	25.00 (47/188)	29.41 (5/17)	1.44	15.95

**Table 8:** Multivariable logistic regression results (odds ratios [95% CI] and p-values) for predicting the need for IMV and mortality & hospice across methods and sites

Outcome	Model	NIV concordance	HFNC concordance	Age	Gender	CCI score	SOFA score	Vent.io score
IMV	RepFlow-CFR	0.678 [0.475.0.968] *p* = 0.032	0.729 [0.497,1.071] *p* = 0.108	0.984 [0.977,0.993] *p* < 0.001	1.021 [0.783,1.333] *p* = 0.877	0.913 [0.869,0.960] *p* < 0.001	1.063 [0.999,1.130]*p* = 0.051	0.836 [0.602,1.160] *p* = 0.284
	LLM-enhanced	0.664 [0.467,0.944] *p* = 0.023	0.679 [0.455,1.012] *p* = 0.057	0.983 [0.974,0.991] *p* < 0.001	1.068 [0.794,1.437] *p* = 0.662	0.904 [0.854,0.958] *p* = 0.001	1.053 [0.982,1.130]*p* = 0.145	0.946 [0.657,1.363] *p* = 0.766
Mortality & hospice	RepFlow-CFR	0.822 [0.589,1.147] *p* = 0.249	0.702 [0.482,1.023] *p* = 0.066	1.015 [1.007,1.023] *p* < 0.001	0.732 [0.566,0.948] *p* = 0.018	0.959 [0.917,1.003] *p* = 0.070	1.312 [1.234,1.396]*p* < 0.001	0.908 [0.663,1.244] *p* = 0.547
	LLM-enhanced	0.716 [0.514,0.997] *p* = 0.048	0.670 [0.451,0.994] *p* = 0.046	1.019 [1.010,1.028] *p* < 0.001	0.685 [0.516,0.909] *p* = 0.009	0.929 [0.882,0.978] *p* = 0.005	1.272 [1.188,1.362]*p* < 0.001	0.851 [0.595,1.218] *p* = 0.377

### Chart review findings

Based on selected chart review by three critical care physicians, as shown in Tables 9 and 95% of LLM recommendations were consistent with the ERS/ATS 2017 guideline for NIV and the ERS 2022 guideline for HFNC. Despite high congruence with the guidelines, physicians overall agreed with the LLM recommendation for only 65% of cases. In the free form comment section provided to reviewers, reasons for disagreement with the LLM included incorrect content in the explanation which was identified in 3 cases (15%). The incorrect content was deemed clinically significant in all 3 cases by critical care physician reviewers. Reviewers also identified missing clinically important information in 6 cases (30%). Of the 11 cases with errors identified in LLM accuracy- either missing important clinical content or incorrect content in the explanation, likelihood of potential harm from these errors was determined to be low in 64% (7/11), medium in 27% (3/11), and high in 9% (1/11) of cases. Potential extent of harm from these errors was determined to be severe/death in 2 cases, mild/moderate in 5 cases and, no harm in 4 cases.

LLM comprehension was excellent, exhibiting correct question comprehension in 100% of reviewed cases. In one case there was both evidence of correct and incorrect question comprehension. The LLM had evidence of correct evidence retrieval and reasoning in 19/20 cases. However, in 6/20 cases the LLM also showed evidence of incorrect retrieval and in 4/20 cases showed evidence of incorrect rationale. Select examples of LLM output and physician review are provided in Supplementary Sect. 4.

**Table 9:** Summary of chart review results for LLM recommendations (*n* = 20)

Domain	Evaluation criteria	*n*	%
**Recommendation congruence**	Is recommendation congruent with the guidelines?	19	95%
**Explanation accuracy**	Is there incorrect content in the explanation?	3	15%
	Is the incorrect explanation clinically significant?	3	15%
	Is the explanation missing important clinical content?	6	30%
**Error harm assessment**	Harm likelihood for LLM error = Low/Med/High/N.A.	Low:7; Med:3; High: 1; N.A.:9	–
	Harm extent from LLM error = None/no harm, Mild/Moderate, Severe/death, N.A.	No harm:4; Mild/Mod:5; Severe/death:2; N.A.:9	–
**Clinical judgment**	Overall MD agreement with LLM recommendation	Yes: 13;No:7; Partially: 0	65%
	First MD agreement	Yes: 17;No: 3; Partially: 0	85%
	Second MD agreement	Yes: 14;No: 5; Partially: 1	70%
	Third MD agreement	Yes: 15;No: 5; Partially: 0	75%
**LLM comprehension & reasoning**	Is comprehension of the question correct?	20	100%
	Is evidence retrieval correct?	19	95%
	Is reasoning for LLM recommendation correct?	Yes:19; No:1; Partially: 0	95%
	Any incorrect comprehension from LLM?	1	5%
	Any incorrect or missing clinical info (retrieval error or hallucination)?	6	30%
	Did the LLM recommendation contain incorrect rationale?	4	20%

## Discussion

We propose and evaluate a framework that couples a deep counterfactual inference model (RepFlow-CFR) with a guideline-aware LLM to generate individualized, guideline-aligned recommendations between HFNC and NIV for acute respiratory failure. Relative to a counterfactual model alone, the LLM component constrains the admissible action space using structured guideline rules and surfaces traceable rationales and deferral logic, improving transparency and clinical plausibility. Associations between concordant care and improved outcomes support the potential clinical utility of knowledge-constrained counterfactual recommendations; given the retrospective design, results should be interpreted as associational and hypothesis-generating.

**Positioning relative to prior work.** Unlike HFNC failure prediction models that focus on patients already receiving HFNC and estimate deterioration within that subgroup, our framework addresses the broader problem of initial modality selection (HFNC vs. NIV) at the time of decision. Beyond existing counterfactual methods which often function as opaque black boxes, we embed guideline constraints via an LLM so that individualized recommendations are clinically admissible, interpretable, and aligned with consensus standards. While retrospective, this study provides a translational foundation for prospective, real-time evaluation of guideline-aware counterfactual decision support in ICU workflows.

### Methodological innovation beyond hybrid approaches

Our framework couples quantitative counterfactual estimates with a guideline-aware correction layer. First, RepFlow-CFR produces individualized potential-outcome estimates and a provisional modality recommendation. The LLM then parses guideline text into structured eligibility/contraindication rules and audits the provisional choice: it (i) blocks recommendations that violate contraindications or minimum criteria, (ii) corrects the recommendation to a guideline-admissible alternative when required, and (iii) defers to a guideline default or clinician review when evidence is weak or data are missing. When multiple actions are admissible, the layer chooses the option with the lower estimated risk from RepFlow-CFR. Each final recommendation is labeled as unchanged/corrected/deferred and exposes the triggering guideline clause(s) plus a brief rationale, linking quantitative estimates to explicit clinical rules.

Conceptually, this acts as a projection of the model’s policy onto the guideline-feasible set, which preserves the counterfactual signal while enforcing clinical admissibility and producing a transparent audit trail. This correction-and-deferral mechanism distinguishes our approach from post-hoc rule displays by actively editing the recommendation when guidelines warrant it, rather than merely annotating it.

### Impact of LLM-guided recommendations on concordance and outcomes

Across recommendation strata, receiving the model-recommended modality was associated with lower IMV and, in several strata, lower Mortality/Hospice. For instance, when the LLM-augmented model recommended NIV, concordant care was associated with lower IMV compared with discordant care; when either modality was recommended, concordant care was associated with lower Mortality/Hospice. Conversely, discordant care was associated with markedly higher IMV risk in some strata, though precision varied with subgroup sizes. Multivariable logistic regression adjusting for age, sex, CCI, SOFA, and Vent.io risk yielded adjusted odds ratios with 95% CIs consistent with these patterns (Table 8). We emphasize effect estimates and confidence intervals over dichotomized significance and treat subgroup contrasts as exploratory.

### Chart review and clinical validity

Structured chart review by critical care physicians confirmed that LLM-generated recommendations were consistent with established guidelines in 95% of cases. However, full clinical agreement with these recommendations was only observed in only 65% of cases, reflecting a critical limitation of guideline-only approaches. Reviewers noted that guidelines alone do not fully capture patient complexity and cannot account for every clinical variable. Examples provided in the supplement highlight several reasons why clinicians disagreed with the LLM which are difficult to capture in ML models, even with guideline driven LLM integration. Several cases involved clinical ambiguity where either HFNC or NIV could be considered acceptable. Additionally, critical care physician reviewers did not agree on the same management approach to all cases, likely reflecting variability in clinical practice patterns. This factor may also pose challenges to implementing LLM-generated recommendations.

Specific patient conditions such as right ventricular failure, hematemesis, or altered mental status were cited as potential contraindications to NIV that were not recognized by the LLM despite technically aligning with guideline criteria. Importantly, the current implementation did not emphasize training the LLM to identify contraindications to NIV or HFNC, nor did it incorporate complex patient-specific modifiers beyond guideline definitions. This reflects a limitation in the structuring of the LLM prompt. However, this approach was intentional for several reasons. First, many of the contraindications to NIV or HFNC require immediate bedside assessment at the time of decision-making and are not readily available in the EHR (e.g. real-time mental status assessment, active hematemesis). Second, the planned future clinical deployment of the model would include a clinical decision support feature that would take clinicians through a list of contraindications that would need to be assessed in real-time to enhance clinical decision making. Although, the contraindications to each specific oxygen modality could potentially be integrated into the LLM, a combined approach of immediate physician assessment plus the ML-LLM best fit the plans for future deployment based on critical care physician input. Such contraindication awareness can be modularly integrated into a decision support tool to better reflect real-world clinical reasoning and improve the robustness of LLM outputs.

### Limitations

Several limitations merit consideration. First, although LLM-guided recommendations improved interpretability and clinical alignment, hallucinations and reasoning errors occurred; in chart review, 30% of cases contained explanatory inaccuracies or omissions, including a smaller subset (15%) with clinically significant issues. Second, guidelines are inherently limited in scope and may not generalize to complex patients with multiple comorbidities. Third, concordance findings are observational and may partly reflect confounding by indication; although multivariable models adjust for age, sex, Charlson Comorbidity Index (which includes a CHF weight), SOFA, and Vent.io risk, residual confounding is possible. Fourth, the single-system cohort limits generalizability. We have initiated application of RepFlow-CFR to the UC Irvine cohort, but integration of clinical notes (needed for guideline-aware LLM recommendations) is ongoing; therefore, external-validation results will be reported separately. Finally, the chart-review sample was small (*n* = 20); larger, prospective evaluations are needed.

### Translation to the bedside and future directions

Our study demonstrates that LLMs can play a powerful role in bridging the gap between black-box machine learning models and interpretable, guideline-adherent clinical decision-making. Future studies will integrate LLM-augmented model recommendations into the EHR, **as** part of an agentic AI system that proactively identifies individuals at risk, and provides real-time recommendations to prevent further clinical decompensation. We will perform prospective implementation in a staged approach that will allow for user-centered design, further model refinement, safety testing and clinician feedback prior to broader implementation. Clinicians will have access to model recommendations at the time of first predicted ARF (from venti.io or from clinical decompensation), and we will measure recommendation-concordance and effect on clinical outcomes over time. Future work will also focus on enhancing the LLM’s ability to detect contraindications, reason about overlapping clinical conditions, and adapt to emerging guidelines. Integrating clinician feedback and deploying this framework prospectively will be essential for developing trustworthy, adaptive AI tools in high-acuity environments such as the ICU.

## Conclusions

We developed and validated a hybrid framework that enhances deep counterfactual inference with LLM-based guideline enforcement to support individualized respiratory support decisions for ICU patients. The LLM-enhanced model improved treatment concordance and was associated with better patient outcomes, including lower rates of IMV and mortality/hospice discharge. While the LLM achieved high guideline adherence, discrepancies with physician judgment highlighted the need to better account for real-world clinical complexity and contraindications. Our findings support the potential of combining explainable AI with evidence-based medicine to build interpretable, high-impact decision support tools in critical care. Further refinement and prospective validation are needed to safely translate this approach into routine clinical practice.

## Supplementary Information

Supplementary Material 1

## Data Availability

The datasets used and/or analysed during the current study are available from the corresponding author on reasonable request.

## Abbreviations

ARF: Acute Respiratory Failure
HFNC: High-Flow Nasal Cannula
NIV: Noninvasive Ventilation
IMV: Invasive Mechanical Ventilation
ICU: Intensive Care Unit
LLM: Large Language Model
ITE: Individualized Treatment Effect
CFR: Counterfactual Regression
CNF: Conditional Normalizing Flow
SOFA: Sequential Organ Failure Assessment
SIRS: Systemic Inflammatory Response Syndrome
CCI: Charlson Comorbidity Index
TSLM: Time Since Last Measurement
EHR: Electronic Health Record
AWS: Amazon Web Services
ED: Emergency Department
H&P: History and Physical
HPI: History of Present Illness
AHRQ: Agency for Healthcare Research and Quality
RCT: Randomized Controlled Trial
OR: Odds Ratio

## References

[1] Arunachala S, Parthasarathi A, Basavaraj CK, et al. The use of high-flow nasal cannula and non-invasive mechanical ventilation in the management of COVID-19 patients: a prospective study. Viruses. 2023;15(9):1879. 37766286 10.3390/v15091879 PMC10535869

[2] Doshi P, Whittle JS, Bublewicz M, et al. High-velocity nasal insufflation in the treatment of respiratory failure: a randomized clinical trial. Ann Emerg Med. 2018;72(1):73-83. e5. 29310868 10.1016/j.annemergmed.2017.12.006

[3] Dugan KC, Hall JB, Patel BK. High-flow nasal oxygen—the pendulum continues to swing in the assessment of critical care technology. JAMA. 2018;320(20):2083–4. 30357275 10.1001/jama.2018.14287

[4] Ferreyro BL, Angriman F, Munshi L, et al. Noninvasive oxygenation strategies in adult patients with acute respiratory failure: a protocol for a systematic review and network meta-analysis. Syst Rev. 2020;9(1):95. 32336293 10.1186/s13643-020-01363-0 PMC7184712

[5] Francio F, Weigert RM, Mattei EDB, et al. High-flow nasal oxygen vs noninvasive ventilation in patients with acute respiratory failure: the renovate randomized clinical trial. JAMA. 2025;333(10):875–90. 39657981 10.1001/jama.2024.26244 PMC11897836

[6] Grieco DL, Menga LS, Cesarano M, et al. Effect of helmet noninvasive ventilation vs high-flow nasal oxygen on days free of respiratory support in patients with COVID-19 and moderate to severe hypoxemic respiratory failure: the HENIVOT randomized clinical trial. JAMA. 2021;325(17):1731–43. 33764378 10.1001/jama.2021.4682 PMC7995134

[7] Linck EJG, Goligher EC, Semler MW, et al. Toward precision in critical care research: methods for observational and interventional studies. Crit Care Med. 2024;52(9):1439–50. 39145702 10.1097/CCM.0000000000006371 PMC11328956

[8] Munroe ES, Prevalska I, Hyer M, et al. High-flow nasal cannula versus noninvasive ventilation as initial treatment in acute hypoxia: a propensity score-matched study. Crit Care Explor. 2024;6(5):e1092. 38725442 10.1097/CCE.0000000000001092 PMC11081605

[9] Nair PR, Haritha D, Behera S, et al. Comparison of high-flow nasal cannula and noninvasive ventilation in acute hypoxemic respiratory failure due to severe COVID-19 pneumonia. Respir Care. 2021;66(12):1824–30. 34584010 10.4187/respcare.09130

[10] Oczkowski S, Ergan B, Bos L, et al. ERS clinical practice guidelines: high-flow nasal cannula in acute respiratory failure. Eur Respir J. 2022. 10.1183/13993003.01574-2021. 34649974 10.1183/13993003.01574-2021

[11] Rochwerg B, Brochard L, Elliott MW, et al. Official ERS/ATS clinical practice guidelines: noninvasive ventilation for acute respiratory failure. Eur Respir J. 2017. 10.1183/13993003.02426-2016. 28860265 10.1183/13993003.02426-2016

[12] Athey S, Wager S. Estimating treatment effects with causal forests: an application. Observational Stud. 2019;5(2):37–51.

[13] Shalit U, Johansson FD, Sontag D. Estimating individual treatment effect: generalization bounds and algorithms. PMLR: 2017.

[14] Künzel SR, Sekhon JS, Bickel PJ, et al. Metalearners for estimating heterogeneous treatment effects using machine learning. Proc Natl Acad Sci U S A. 2019;116(10):4156–65. 30770453 10.1073/pnas.1804597116 PMC6410831

[15] Chipman HA, George EI, McCulloch RE. BART: bayesian additive regression trees. Ann Appl Stat. 2010;4:266–98.

[16] Lam JY, Lu X, Shashikumar SP, et al. Development, deployment, and continuous monitoring of a machine learning model to predict respiratory failure in critically ill patients. JAMIA Open. 2024. 10.1093/jamiaopen/ooae141. 39664647 10.1093/jamiaopen/ooae141 PMC11633942

[17] Winkler C, Worrall D, Hoogeboom E et al. Learning likelihoods with conditional normalizing flows. ArXiv Preprint arXiv:191200042 2019.

[18] Singhal K, Azizi S, Tu T, et al. Large language models encode clinical knowledge. Nature. 2023;620(7972):172–80. 37438534 10.1038/s41586-023-06291-2 PMC10396962

[19] Anthropic. (2024). Claude 3.5 Sonnet [Large language model]. https://claude.ai

