# Supplementary material for: Enhancing predictive modeling for respiratory support with LLM-driven guideline adherence
Source: Crit Care. 2025 Nov 14;29:491. doi: 10.1186/s13054-025-05739-3 (PMC12619420; doi:10.1186/s13054-025-05739-3)
Supplement: Supplementary file 1 — Supplementary Material 1 [file 13054_2025_5739_MOESM1_ESM.docx]

**Supplementary Materials for “Enhancing Predictive Modeling for Respiratory Support with LLM-Driven Guideline Adherence”**

Section 1: EHR features used for the RepFlow-CFR model.

Table 1. Model features summary.

| **Demographic features** | Age, Gender, care unit identifier - ED, care unit identifier- Ward, hours between hospital admit and care unit admit (ED/Ward), duration from care unit admission until current time |
| --- | --- |
|  |  |
| **Vital signs and laboratory measurements** | HR, O2Sat, Temp, SBP, MAP, DBP, Resp, EtCO2, BaseExcess, HCO3, FiO2, pH, PaCO2, SaO2, AST, BUN, Alkalinephos, Calcium, Chloride, Creatinine, BilirubinDirect, Glucose, Lactate, Magnesium, Phosphate, Potassium, BilirubinTotal, TroponinI, Hct, Hgb, PTT, WBC, Fibrinogen, Platelets, PaO2, BNP, Sodium, Procalcitonin, CRP, Ammonia, Hgb_A1C, lymphocytes, lymphocytes_differential, albumin, ALT, D_Dimer, Ferritin, Red_cell_width, Sedimentation_Rate, LDH |
| **Comorbidities** | Disseminated intravascular coagulation, Necrotizing Fasciitis, Tumor Lysis Syndrome, Shock, Metastatic Cancer, STEMI, Myeloblastic Leukemia, Liver Failure, Malignant Pleural Effusion, Pneumothorax, Cachexia, Coma, Peritonitis, Lymphoma, Endocarditis, Myocarditis, Pericarditis, Septic Embolism, Coagulation Defect, Primary Lung Cancer, Lymphoid Leukemia, Pericardial Effusion, Cerebral Infarction, Meningitis/Encephalitis, Malignant Liver Cancer, GI Hemorrhage, Acute Renal Failure, Chronic Liver Disease, Neutropenia, Cardiac Arrhythmia, Malnutrition, Pulmonary Heart Disease, Chronic Kidney Disease, Plasma Protein Disorder, Pancytopenia/Aplasia, Aspiration Pneumonitis, AMI/Non-STEMI, CHF, Encephalopathy, Thrombocytopenia Including Purpuric, HIT, & Other Platelet defects, Coronary artery disease, COPD, Diabetes, HIV, Hypertension, Obesity, Severe Brain Conditions, Other Pulmonary Conditions, Non-Rheumatic Valve Disease, UTI, Anemia, Pneumonia, Hyperlipidemia, Major depressive disorder, Bacterimia, Gastro-esophageal reflux disease, Hypothyroidism, Long term use of insulin, Liver cirrhosis, Immune conditions, Solid malignancy, Organ transplant, Rheumatologic/Inflammatory |
| **Medications** | Anesthesia, Anticoagulants, Anticonvulsants, Antipsychotics, Bleeding reversal agents, Continuous infusion vasodilator, Continuous infusion anti-hypertensive, Continuous infusion antiarrhythmics, Inhaled prostacyclin, Continuous infusion for neuromuscular blockade, Continuous infusion for pain, Steroids |

**Section 2: RepFlow-CFR Development, Training and Evaluation**

We proposed the RepFlow-CFR model, a flow-based confounder adjustment model that integrates representation learning, normalizing flows and counterfactual inference. Figure 1 presents the architecture overview of the RepFlow-CFR model, which contains three modules as follows.

Stage 0 (CFR-based representation learning): We utilized the counterfactual regression architecture (CFR) that includes shared representation layers and two distinct heads for predicting outcomes under different treatments. The shared representation layers, based on the Vent.io architecture, include a TSLM layer for adjusting the importance of labs and vitals, followed by a feedforward neural network. By training the CFR model, the shared representation is encouraged to balance the distribution of measured confounders across treatment groups. The loss function is formulated as

$L_{0}=E\left[ L_{CFR}\left( x,a,y \right)+\lambda\cdot IPM_{G} \left( \left\{ \phi_{i} \right\}_{i,a=HFNC} ,\left\{ \phi_{i} \right\}_{i,a=NIV} \right) \right]$,

where $IPM_{G}$ is the empirical probability metric (e.g. Wasserstein distance). $L_{CFR}$ is the prediction loss. $\lambda$ denotes the trade-off parameter that balances prediction accuracy and representation distribution matching.

After training Stage 0, we assume that the learned representation $\phi$ captures sufficient information from measured covariates such that potential outcomes are conditionally independent of treatment assignment, given $\phi$ and a latent variable $u$ representing unmeasured confounding as

$$Y\left( A \right) ⫫A |\phi,u,$$

where $Y$ and $A$ denote the outcome and treatment, respectively.

The observed distribution is defined as

$p\left( y | \phi,a \right)=\int p(y|\phi,u,a)p(u|\phi,a)du$.

The interventional distribution, which removes the influence of $a$ on $u$, is

$p\left( Y\left( a \right)=y | \phi\right)=\int p(y|\phi,u,a)p(u|\phi)du$.

If $p\left( u | \phi,a \right)= p\left( u | \phi\right),$we will have $p\left( Y\left( a \right)=y | \phi\right)=p\left( y | \phi,a \right)$. However, in observational studies, this assumption rarely holds due to treatment assignment bias or unmeasured confounding. Directly using $p\left( y | \phi,a \right)$ to estimate counterfactual outcomes would lead to biased inference. To address this, RepFlow-CFR includes two additional stages to explicitly model and account for this hidden bias.

Stage 1 (Modeling outcome distribution): We used a conditional normalizing flow (CNF) to model the observed outcome distribution $p\left( y | \phi,a \right)$, where $a\in\left\{ NIV, HFNC \right\}$. The CNF learns an invertible transformation $f_{\phi,a}^{1}$ that maps a standard normal latent variable $U\sim N(0,I)$ to the outcome space. The loss function is formulated as

$$L_{1} =\sum_{i=1}^{n} p\left( f_{\phi_{i},a_{i}}^{1}\left( U \right)=y_{i} \right).$$

Stage 2 (Adjusting for hidden confounding): Since the learned representation $\phi$ may not satisfy unconfoundedness due to unmeasured confounding, we introduced a second CNF $f_{\phi,a}^{2}$ . It transforms a new latent variable $\tilde{U}\sim N(0,I)$ to an interventional latent variable that approximates $p(u|\phi)$. The resulting latent sample is then passed through the Stage 1 transformation

$f_{\phi,a}^{1}$, which shifts the latent distribution $p\left( u | \phi,a \right)$ toward the interventional distribution $p(u|\phi)$. This adjustment allows us to account for hidden biases arising from factors like clinician decision-making, treatment selection bias, and unobserved patient severity. The loss function is formulated as

$L_{2}=\sum_{i=1}^{n} p\left( f_{\phi_{i},a_{i}}^{1}\left( f_{\phi_{i},a_{i}}^{2}(\tilde{U}) \right)=y_{i} \right)$.

Inference stage: During inference, we sampled $\tilde{U}$ using the CNF from Stage 2 and mapped it to $y$ using the CNF from stage 1, then averaged multiple predicted outcomes. ITE was obtained by the difference between the averaged outcomes under NIV and under HFNC.

**Inference.** During inference, we sample $\tilde{U}$via Stage 2 and map it through Stage 1 to obtain potential outcomes under both HFNC and NIV; multiple draws are averaged for stability. The individual treatment effect (ITE) is the difference in predicted IMV probabilities under NIV versus HFNC. Treatment recommendations follow: NIV preferred (ITE < −0.001), HFNC preferred (ITE > 0.001), or Indifferent (−0.001 ≤ ITE ≤ 0.001).

**Training and evaluation.** The UCSD early HFNC/NIV cohort was split 80%/20% into training/validation. We applied Bayesian optimization to tune hyperparameters including learning rate, weight regularization, number of hidden layers, flow depth, dropout rate, and batch size; the process was guided by validation performance across the CFR module and both CNF modules. Each module was trained separately using the Adam optimizer, with early stopping based on validation loss to mitigate overfitting. To ensure robustness at inference, we drew 100 latent samples per encounter from Stage 2 and mapped them through Stage 1; predictions were averaged to produce stable estimates for each treatment condition. Model performance—including discrimination (AUC, PR-AUC) and ITE-estimation quality—was evaluated on the UCSD cohort. **The complete hyperparameter search space and the selected settings are listed in Tables 2.**

**Table 2. Hyperparameter search space (Bayesian optimization).**

| **Hyperparameter** | **Search space (from code)** | **Notes** |
| --- | --- | --- |
| **CFR: number of hidden layers** | {2, 3, 4} | sampled uniformly at each Bayesian optimization call |
| **CFR: hidden layer width** | {50, 55, 60, …, 150} | step = 5 (from 50–150) |
| **CFR: width decay factor** | {0.5, 0.7, 0.9} | derived from 0.5–0.9 with step 0.2 |
| **CNF (Stage-2): number of hidden layers** | {2, 3, 4} | sampled uniformly |
| **CNF (Stage-2): hidden layer width** | {50, 55, 60, …, 150} | step = 5 |
| **CNF (Stage-2): width decay factor** | {0.5, 0.7, 0.9} | step 0.2 |
| **CFR: λ₁, λ₂ (L2/etc.)** | {1e-3, 1e-4, 1e-5, 1e-6} |  |
| **CNF (Stage-2): λ₁, λ₂** | {1e-3, 1e-4, 1e-5, 1e-6} |  |
| **Learning rate** | {1…9}×{10^−2^,10^−3^,10^−4^} |  |
| **Dropout rate** | {0, 0.10, 0.15, 0.20} | sampled uniformly per call |
| **Bayesian optimization calls** | n_calls = 200 | gp_minimize |


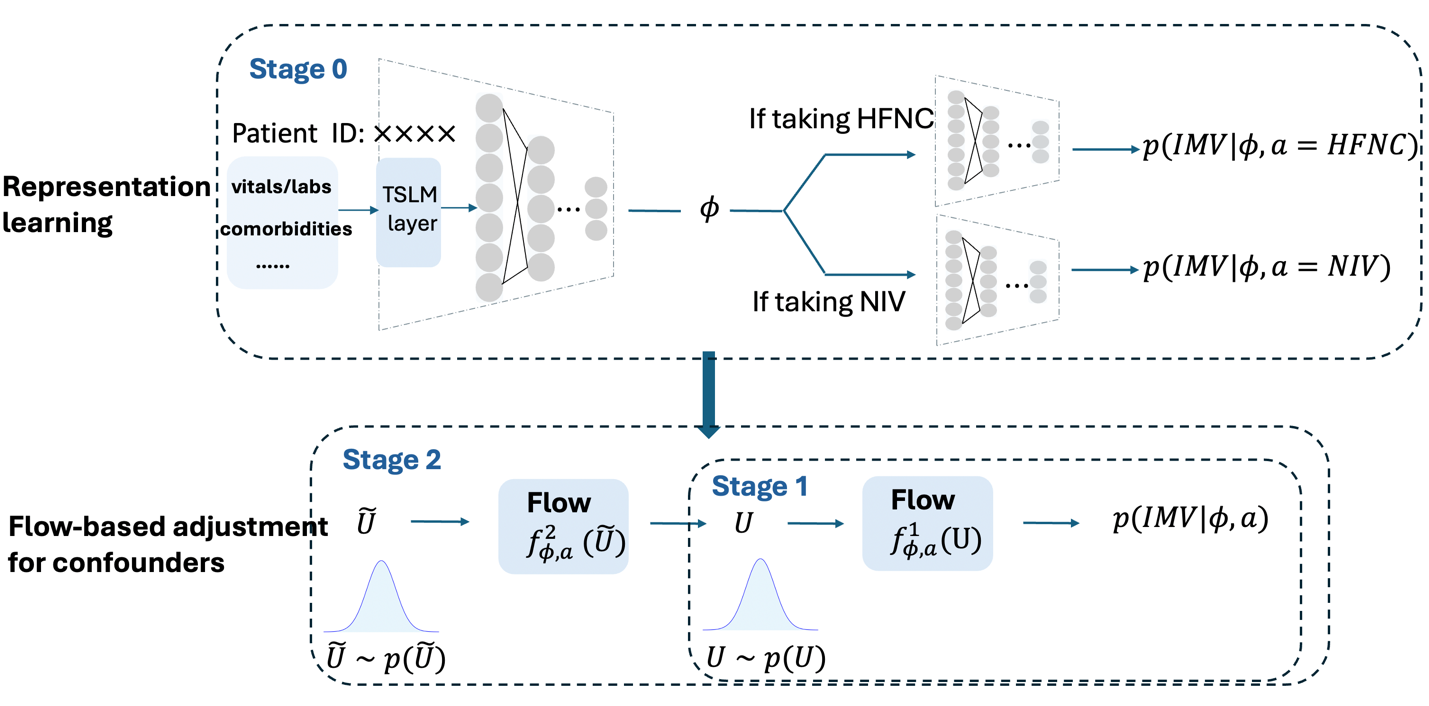


Figure 1. Overview of the RepFlow-CFR model, which contains CFR-based representation learning, output distribution modeling and hidden confounding adjustment.

Section 3: Instruction prompt for the LLM.

Table 2. Instruction prompt.

| **prompt** = """You are a clinical reasoning assistant. You will be provided with:  - **The RepFlow-CFR model recommendation**, including:  (1) the preferred oxygenation modality (NIV or HFNC or Indifferent), and  (2) the rationale based on EHR data.  - **Recent clinical information** (EHR, notes and radiology report), particularly from the last 24–48 hours.  **Your tasks:**  1. Review all details provided in the <context> tags carefully.  2. Compare the RepFlow-CFR recommendation with current clinical guideline criteria.  3. Determine whether the model's recommendation is aligned with guideline-based decision rules.  4. Make an independent recommendation based solely on the clinical data using the guideline criteria (NIV, HFNC, or Indifferent).  5. Provide rationale citing relevant parts of the clinical guideline.  6. If data is insufficient, return: `"insufficient_data": true`.  **### Guideline Criteria Summary:**  **### NIV-ERS/ATS 2017 Guidelines:**  Recommend NIV if any of the following are true:  - COPD exacerbation with acute or acute-on-chronic respiratory acidosis (pH ≤ 7.35, PaCO₂ > 45 mmHg)  - Acute respiratory failure due to cardiogenic pulmonary edema, including acute (or “flash”) pulmonary edema  - Neuromuscular disease or obesity hypoventilation syndrome (OHS) with acute or acute-on-chronic respiratory acidosis (pH ≤ 7.35, PaCO₂ > 45 mmHg)  - Use NIV prophylatically post-extubation in high-risk patients (for example, those with COPD or CHF) but not in low risk patients or in patients with established post-extubation respiratory failure  - Immunocompromised patients with mild-to-moderate acute respiratory failure (conditional recommendation).  - post-operative ARF. (Conditional recommendation, moderate certainty of evidence.)  - chest trauma patients with ARF. (Conditional recommendation, moderate certainty of evidence.)  **### HFNC ERS 2022 Guidelines**  Recommend HFNC if any of the following are true:  - De novo (or acute) hypoxemic respiratory failure (e.g., AHRF, ARDS, pneumonia, COVID-19)  - Post-operative post-extubation in high-risk patients: HFNC is preferred over conventional oxygen but NIV is also an acceptable prophylactic treatment for high-risk patients.  - The patient isintolerant to NIV and moderate to severe acute respiratory failure is present  - Moderate to severe acute respiratory failure is present and there is not an indication for treatment with NIV.  Do not recommend HFNC as first-line if:  - The patient has acute hypercapnic respiratory failure (e.g., COPD with acidosis) unless NIV is contraindicated or not tolerated.  - There is clear guideline indication for NIV  **Suggested JSON Output Format**  Below is an example of how you might structure your response in JSON.  {  "o2_recommendation":{  "niv_recommended": {"answer": "Yes or No or Either",  "confidence": "high / medium / low",  "explanation": "Brief rationale"  },  "hfnc_recommended": {"answer": "Yes or No or Either",  "confidence": "high / medium / low",  "explanation": "Brief rationale"  }  "model_alignment": {  "alignment": "true / false",  "explanation": "Was RepFlow-CFR aligned with guideline-based decision?"  }  }  }  """  # Define the JSON schema  json_format = [{  "toolSpec": {  "name": "o2_recommendation",  "description": "Return a structured JSON recommendation for NIV vs. HFNC based on the patient summary.",  "inputSchema": {  "json": {  "type": "object",  "properties": {  "niv_recommended": {  "type": "object",  "properties": {  "recommendation": {  "type": "string",  "description": "niv_recommended",  "enum": ["Yes", "No", "Either"]  },  "confidence": {  "type": "string",  "description": "Certainty assessment based on the available documentation. ",  "enum": ["high", "medium", "low"]  },  "explanation": {  "type": "string",  "description": "Brief rationale including guideline reference."  }  }  },  "hfnc_recommended": {  "type": "object",  "properties": {  "recommendation": {  "type": "string",  "description": "hfnc_recommended",  "enum": ["Yes", "No", "Either"]  },  "confidence": {  "type": "string",  "description": "Certainty assessment in based on the available documentation. ",  "enum": ["high", "medium", "low"]  },  "explanation": {  "type": "string",  "description": "Brief rationale including guideline reference."  }  }  },  "model_alignment": {  "type": "object",  "properties": {  "alignment": {  "type": "string",  "description": "model_alignment",  "enum": ["true", "false"]  },  "explanation": {  "type": "string",  "description": "Was RepFlow-CFR aligned with guideline-based decision?"  }  }  },    },    "required": [  "niv_recommended",  "hfnc_recommended",  "model_alignment",    ]  }  }  }  }] |
| --- |

Section 4: LLM- enhanced model output examples and critical care physician agreement/disagreement with summary of physician comments

**Case example 1: physician disagreement with LLM and example of missed contraindications**

LLM recommendation and rationale: Patient has clear evidence of cardiogenic pulmonary edema per latest CXR showing 'persistent and slightly worsening perihilar congestion and edema' with 'cardiogenic' etiology. This meets ERS/ATS 2017 guideline criteria for NIV in acute respiratory failure due to cardiogenic pulmonary edema. Per ERS 2022 guidelines, HFNC is not recommended as first-line when there is a clear guideline indication for NIV, which exists here with cardiogenic pulmonary edema.

Physician #1: Disagree

Physician #1 comments: Patient coughing up blood, hematemesis, significant secretions, and documented to not tolerate NIV.

Physician #2: Disagree

Physician #2 comments: disagree due to contraindications- hemoptysis, secretions, not tolerating NIV.

Physician #3: Disagree

Physician #3 comments: Disagree due to NIV contraindications.

Harm extent from LLM error/disagreement: Severe/death

**Case example 2: physician disagreement with LLM and example of variability in practice patterns**

LLM recommendation and rationale: Patient has acute pulmonary edema (confirmed by radiology) with signs of respiratory distress (RR 28). Patient also has chronic systolic heart failure. Per ERS/ATS 2017 guidelines, NIV is strongly recommended for acute cardiogenic pulmonary edema. Given the clear indication for NIV (acute pulmonary edema in a patient with CHF), HFNC would not be first-line per ERS 2022 guidelines which state not to use HFNC when there is a clear guideline indication for NIV.

Physician #1: Disagree

Physician #1 comments: Disagree due to altered mental status is a contraindication.

Physician #2: Agree

Physician #2 comments: Although patient appears very agitated, would have to see if patient would tolerate NIV perhaps with the aid of dexmedetomidine drip.

Physician #3: Disagree

Physician #3 comments: LLM recommends NIV which I agree is indicated, however notes report encephalopathy so would do HFNC due to patient’s intolerance.

Consensus statement: (Disagree) After consensus review, physicians disagreed with LLM. Disagree with LLM and prefer HFNC due to severe altered mental status. Although NIV is appropriately indicated based on pathology, would use HFNC first if patient was too agitated.

**Case Example #3: physician disagreement with LLM with example of clinical equipoise**

LLM recommendation and rationale: Patient has de novo hypoxemic respiratory failure without clear indications for NIV per ERS/ATS 2017 guidelines. No evidence of COPD exacerbation, cardiogenic pulmonary edema, or neuromuscular disease. SpO2 93% with tachypnea (RR 52) suggests acute hypoxemic respiratory failure. Per ERS 2022 guidelines, HFNC is indicated for de novo hypoxemic respiratory failure. Patient has tachypnea (RR 52) and hypoxemia (SpO2 93%) without clear NIV indications. Likely related to metastatic cancer with possible pulmonary involvement.

Physician #1: Disagree

Physician #1 comments: Either NIV or HFNC for work of breathing would be ok for this patient. Recommendation should be that both options are ok. It makes sense to say no NIV indication from the guideline and some hypoxia so use HFNC but bit picture is that HFNC and NIV are mainly treating work of breathing here and either modality would be acceptable. The guidelines do not capture this type of patient complexity in my opinion.

Physician #2: Disagree

Physician #2 comments: Either HFNC or NIV would be appropriate

Physician #3: Disagree

Physician #3: Either NIV or HFNC would be ok.

**Case #4: Physician disagreement with LLM and example of incorrect rationale/laboratory interpretation**

LLM recommendation and rationale: Patient has severe COPD with acute respiratory failure. pH of 7.33 indicates respiratory acidosis, with clinical signs of respiratory distress (RR 28, increasing O2 requirements). Per ERS/ATS 2017 guidelines, NIV is strongly recommended for COPD exacerbation with acute respiratory acidosis. Per ERS 2022 guidelines, HFNC is not recommended as first-line therapy in acute hypercapnic respiratory failure, especially in COPD patients with acidosis unless NIV is contraindicated/not tolerated. There are no contraindications to NIV noted.

Physician #1: Agree

Physician #1 comments: Complex patient with COPD, increased work of breathing (so NIV ok), but also fevers, leukocytosis, possible decompensated right ventricular failure. Would worry about right ventricle here. Also incorrect interpretation of blood gas, no acute respiratory acidosis. Either therapy (HFNC or NIV) may be appropriate, challenging to tell with multiple processes occurring.

Physician#2: Disagree

Physician #2 comments: Would have tried HFNC first, Not hypercapnic like LLM mentioned and no pulmonary edema, has right ventricular dysfunction.

Physician #3: Agree

Physician #3: Mixed disease, COPD with acute hypoxemic respiratory failure, with possible cardiogenic component and right ventricular dysfunction. NIV or HFNC may be appropriate. NIV ok as first strategy.

Consensus statement: (Disagree) HFNC would be recommended. LLM interpretated acute hypercapnic respiratory failure wrong. Even in the setting of increased work of breathing this does not mean 100% that it should be NIV. Also with right ventricular dysfunction potentially NIV is dangerous.
